# Regional height growth models for Scots pine in Poland

**DOI:** 10.1038/s41598-021-89826-9

**Published:** 2021-05-14

**Authors:** Jarosław Socha, Luiza Tymińska-Czabańska, Karol Bronisz, Stanisław Zięba, Paweł Hawryło

**Affiliations:** 1 Department of Forest Resources Management, Faculty of Forestry, University of Agriculture in Krakow, Al. 29 Listopada 46, 31-425 Krakow, Poland; 2 Institute of Forest Sciences, Warsaw University of Life Sciences-SGGW, Nowoursynowska 159, 02-776 Warsaw, Poland

**Keywords:** Ecology, Ecological modelling

## Abstract

Site productivity remains a fundamental concern in forestry as a significant driver of resource availability for tree growth. The site index (SI) reflects the overall impact of all environmental factors that determine tree height growth and is the most commonly used indirect proxy for forest site productivity estimated using stand age and height. The SI concept challenges are local variations in climate, soil, and genotype-environmental interactions that lead to variable height growth patterns among ecoregions and cause inappropriate estimation of site productivity. Developing regional models allow us to determine forest growth and SI more appropriately. This study aimed to develop height growth models for the Scots pine in Poland, considering the natural forest region effect. For height growth modelling, we used the growth trajectory data of 855 sample trees, representing the Scots pine entire range of geographic locations and site conditions in Poland. We compared the development of regional height growth models using nonlinear-fixed-effects (NFE) and nonlinear-mixed-effects (NME) modelling approaches. Our results indicate a slightly better fit to the data of the model built using NFE approach. The results showed significant differences between Scots pine growth in natural forest regions I, II, and III located in northern Poland and natural forest regions IV, V, and VI in southern Poland. We compared the development of regional height growth models using NFE and NME modelling approaches. Our results indicate a slightly better fit to the data of the model built using the NFE approach. The developed models show differences in height growth patterns of Scots pines in Poland and revealed that acknowledgement of region as the independent variable could improve the growth prediction and quality of the SI estimation. Differences in climate and soil conditions that distinguish natural forest regions affect Scots pine height growth patterns. Therefore, extending this research to models that directly describe height growth interactions with site variables, such as climate, soil properties, and topography, can provide valuable forest management information.

## Introduction

Site productivity remains a fundamental concern in forestry as a significant driver of resource availability^1^. Appropriate estimation of site productivity is critical for forest management decisions^2^. Additionally, information on site productivity helps monitor and assess climate change impact on forest ecosystems and provide information to support forest management to take effective adaptation measures^3^. The site index (SI) reflects the overall impact of all environmental factors that determine tree height growth^4^ and is the most commonly used indirect proxy for forest site productivity estimated using stand age and height^2,5–9^. An appropriate height growth model is needed to determine the SI accurately.

One of the most critical challenges in the SI concept is a local variation of height growth patterns that result from a variety of factors and could cause the under- or over-estimation of the growth potential and productivity of a site^10,11^. Species variability caused by sensitivity to environmental factors is the primary reason why a more flexible approach to developing height growth models is recommended to include the impact of local conditions and to accurately reflect height growth variation on a regional scale^12^.

Climate, soil, and genotype-environmental interactions can favour species adaptation to local ecological conditions, causing variable height growth patterns for the same species among ecoregions^10,11^. In a study conducted in Sweden, Johansson^13^ found significant variation in forest height growth curves due to mineral soil type and geographical location. The effect of genetic variability on the height development of trees was reported by Adams et al.^14^. Genetic variability has also been identified as the primary factor modifying the trajectory of height growth by Buford and Burkhart^15^, who found that the height growth models developed for different provenances differed in their parameters describing the asymptote. Significant differences in height growth patterns can also occur for the same species, even within a given SI class^16^. García^17^ reported that three sources of height growth development variability could be identified: (a) between sites, (b) within sites, and (c) observation error. Because of the inter-regional variability of growth patterns, regional height growth models (RMs) are derived to improve the model's predictive ability^11,12,18–20^. Studies on Pinus pinaster in Spain indicated significant differences in growth patterns observed in different areas because of the effect of genotype-environment interaction^11^. Research describing regional height growth differences highlights the regional application of the SI concept^18,21–24^. The role of regional models may be crucial for sustainable forest management, particularly in solving problems that require accurate information on local growing conditions, where climate and soil type lead to inter-regional variation^20^.

**Figure 1:**
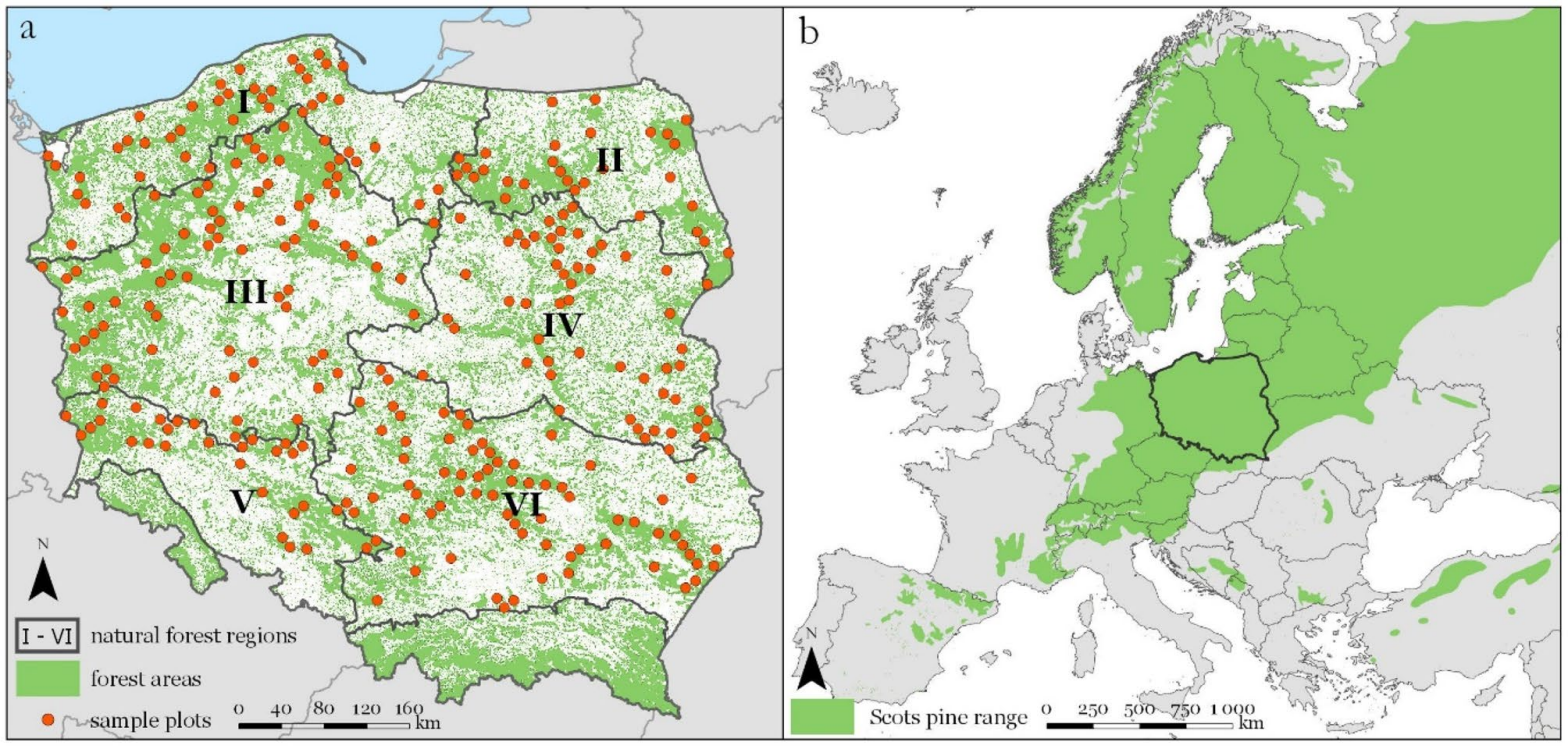
Natural forest regions in Poland with locations from which stem analysis data were collected (**a**); the range of Scots pine distribution in Europe (**b**). The map was generated using ArcGIS software^35^ (ArcGIS Pro, Version 2.2.0, https://www.esri.com/en-us/arcgis/products/arcgis-pro/overview). The Scots pine range shapefile published by Caudullo et al.^36^ under the CC BY 4.0 open-access license was the basis for the mapping.

The Scots pine is one of the most important species in Europe and ranges from the boreal region in Northern and Eastern Europe to the mountains of the Mediterranean in Southeastern Europe (Fig. 1). It is also the most important tree species in Poland, covering approximately 58% of the total forested area of the country^25^. The Scots pine grows under very variable ecological conditions^26^, which could affect the difference in the shape of the growth curves among ecoregions^13^. The effective management of such a considerable forest resource requires a thorough assessment of the productivity of stands at a regional level^27^. Growth modelling is crucial in assessing the consequences of forest management activities in long term planning in forestry^28^. The use of inappropriate height growth models may result in erroneous estimation of site productivity. This problem could be solved by the development of models, which reflect the difference in growth trajectories resulting from specific growth conditions in a given area. Regional models reflect the impact of regional variability on height growth dynamics resulting from specific climate conditions, soil properties, and differences in local forest management.

In Poland, yield tables with discrete scales are still used in forest management for forest growth and site productivity estimation^29–31^. The forestry practice reports the incorrect determination of site productivity and incremented volume using yield tables, probably because of the inadequacy of Schwappach's tables from differences in growth conditions, which have changed considerably during the 100 years since the yield tables were developed. Pretzsch et al.^32^ estimated that in Central Europe, site productivity increased by approximately 30–70%. Additionally, the height growth of observed stands differs significantly from that observed 100 years ago^33,34^ and included in the original tables. Moreover, the tables produce errors by not considering regional growth variability on a countrywide scale.

The research adopted the hypothesis that height growth is regionally differentiated. We assumed that height growth patterns are influenced by the variability in site conditions, which can be acknowledged by considering the specific effect of natural forest regions (NFE), distinguished by environmental conditions. Therefore, the research objective was to analyse the variability of height growth patterns between natural forest regions in Poland and develop height growth models for the Scots pine in Poland taking into account the specific effect of the region.

## Materials and methods

### Collection of the research material

The research material consists of the height growth trajectories of 855 dominant and codominant Scots pines (Table 1). The most substantial part of growth trajectories was collected from the sample plots established within the project "Actual and potential site productivity in Poland for main forest-forming tree species" from 2015 to 2016 and representing the whole variability of growth conditions in Poland for the Scots pine trees. Other tree growth trajectories have been collected in Poland over the last 2 decades as part of research projects conducted by the Department of Forest Resources Management, University of Agriculture, Krakow, the Forest Research Institute in Sękocin Stary, and the Faculty of Forestry, Warsaw University of Life Sciences. The final set of sample plots represent the area of Poland, with a whole range of geographic distributions and site conditions (Fig. 1). The age of the analysed sample trees ranged from 12 to 175 years, with an average of 76 years and heights ranging from 3.50 to 36.75 m (Table 1).

**Table 1:** The number and basic characteristics of growth series for the Scots pine trees, obtained from stem analysis and used for the development of the site index models.

Natural forest region	Number of growth series	Age (years)			Height (m)		
		Mean	Min	Max	Mean	Min	Max
I	146	74	20	140	21.2	13.2	33.4
II	125	88	12	175	28.2	7.1	36.7
III	174	75	14	175	20.6	8.2	33.8
IV	60	68	17	112	23.9	9.9	33.2
V	39	63	12	145	21.8	3.5	33.1
VI	311	75	23	141	23.3	11.0	35.6
Total	855						

Natural forest regions^37^ were adopted as the regionalisation unit in Poland. Natural forest regions were distinguished by the natural diversity of the country, in particular: variability of climatic and geological conditions, naturally occurring ranges of principal forest-forming tree species, presence and distribution of natural landscapes classes, and distribution of the primary units of potential natural vegetation^37^.

### Stem analysis

Stem analysis was used to reconstruct the past height growth of the trees. The trees for stem analysis were selected in the direct neighbourhood of the permanent sample plots established in Scots pine stands. For each plot, tree diameter and height close to the top height (TH)—defined as the average height of the 100 thickest trees per 1 ha—were selected and cut for stem analysis (SA). Only trees with typically developed crowns and no visible damage or growth anomalies were selected for SA. After cutting, the total lengths of the trees were measured, and discs were collected for SA. Discs were taken from the base of the felled tree at the height of 0.5 m, and the breast height diameter (DBH) was measured; for trees over 15 m high, subsequent discs were collected at heights of 2.0, 4.0, and successive 2-m intervals to the top of the tree. For trees shorter than 15 m, subsequent discs were collected at heights of 1.5, 2.5, 3.5, and at 1-m intervals further up the tree. The course of growth of the individual trees was reconstructed based on the height of the discs and the number of annual discs. The growth trajectories of all collected trees were visually examined for suppression and release patterns and growth anomalies. As cross-section lengths do not coincide with periodic height growth, the height-age data from the SA were corrected using Carmean's method^38^. The collected growth series for six natural forest regions were used to develop regional height growth models for Scots pine.

### Models and parameters estimation

To develop regional height growth models: nonlinear fixed-effects and nonlinear mixed-effects modelling approaches were used.

#### A nonlinear mixed-effects modelling approach

The growth series are characterised by a hierarchical structure, which contains information about individual trees and natural forest regions. The mixed-effects modelling approach is a possible solution to handle such data^39–42^. To develop the height growth model using a mixed-effects modelling approach, we tested three functions well known in forest growth modelling: Chapman–Richards, Schumacher and Hossfeld:

1 $${\text{Chapman{-}Richards:}}\quad {\text{H}} = {\text{a}}_{1} \left( {1 - {\text{e}}^{{{\text{a}}_{2} {\text{t}}}} } \right)^{{{\text{a}}_{3} }}$$

2 $${\text{Schumacher:}}\quad {\text{H}} = {\text{e}}^{{{\text{a}}_{1} + {\text{a}}_{2} {\text{t}}^{{{\text{a}}_{3} }} }}$$

3 $${\text{Hossfeld:}}\quad {\text{H}} = \left( {\frac{{{\text{a}}_{1} {\text{t}}^{{{\text{a}}_{2} }} }}{{{\text{a}}_{3} + {\text{t}}^{{{\text{a}}_{2 - 1} }} }}} \right)^{1/3}$$

where *H* is the top height (m), *t* is an age, and *a*_*1*_*-a*_*3*_ are estimated parameters.

Given the value of the mean absolute error (MAE), adjusted coefficient of determination (${\text{R}}_{{{\text{adj}}}}^{2}$), and the Akaike information criterion (AIC), the possibility of defining different random parameters was achieved, with a maximum of all (three) parameters found in the tested functions on both: trees and natural forest regions as grouping levels. In the case of heteroscedastic data, analysing likelihood ratio test and solutions containing power-type variance function with age as a predictor were applied^43^. Furthermore, given within-tree autocorrelation, the autoregressive correlation structure of order 1—AR(1)^44^ and autoregressive–moving average models for the within-group errors was analysed using *corAR1* and *corARMA(10,0)* function from *nlme* R package respectively^41^ with RStudio software^45^.

#### Development of the models using nonlinear fixed-effect modelling approach with the functions developed using the algebraic difference approach

The algebraic difference approach (ADA) formulated by Bailey and Clutter^46^ allows for the derivation of models in which the SI depends only on one parameter. Therefore it allows obtaining polymorphic models with single asymptotes or anamorphic models with multiple asymptotes. The generalised algebraic difference approach (GADA) developed by Cieszewski and Bailey^47^ allows the use of multiple site parameters and models characterised by polymorphism and variable asymptotes for different sites. Height growth models should also show equality of the SI and height at base age and use the same function as the height growth and SI models.

After initial preselection, three ADA models (M2, M4 and M5) and two GADA models (M1, M3) were chosen for further analysis (Table 2). These models meet the criteria mentioned above and have been successfully applied in SI modelling, making them potential candidates for reliable SI models.

**Table 2:** The dynamic generalised algebraic difference approach (GADA) and algebraic difference approach (ADA) formulation of growth functions tested in height growth model development.

Model no	Base model forms	Parameter related to site	Solution for theoretical variable X	Dynamic ADA or GADA formulation and reference
M1	$H = \frac{{a_{1} S^{{a_{2} }} }}{{\left( {1 + e^{{a_{3} - a_{4} lnt - a_{5} lnS}} } \right)}}$	$b = b_{1} + X$ $a = a_{1} /X$	$R = Z_{0} + \left( {Z_{0}^{2} + \frac{{2b_{2} H_{0} }}{{T_{0}^{{b_{1} }} }}} \right)$ $Z_{0} = H_{0} - b_{3}$	$H_{1} = H_{0} \frac{{T_{1}^{{b_{1} }} (T_{0}^{{b_{1} }} R + b_{2} )}}{{T_{0}^{{b_{1} }} \left( {T_{1}^{{b_{1} }} R + b_{2} } \right)}}$^48^
M2	$H = a_{1} \left[ {1 - \exp \left( { - a_{2} T} \right)} \right]^{{a_{3} }}$	$a_{1} = X$	$X_{0} = H_{0} /\left( {1 - {\text{exp}}( - b_{2} T_{0} } \right))^{{b_{3} }}$	$H_{1} = H_{0} \left( {\frac{{1 - exp^{{ - b_{2} T_{0} }} }}{{1 - exp^{{ - b_{2} T_{1} }} }}} \right)^{{b_{3} }}$^49^
M3	$H = a_{1} \left[ {1 - \exp \left( { - a_{2} T} \right)} \right]^{{a_{3} }}$	$a_{1} = exp\left( X \right)$ $a_{3} = b_{2 + } b_{3} /X$	$X_{0} = \frac{1}{2}lnY_{0} - b_{2} L_{0} + \sqrt {\left( {lnY_{0} - b_{2} L_{0} } \right)^{2} - 4b_{3} L_{0} }$ with $L_{0} = ln1 - \exp \left( { - b_{1} T_{0} } \right)$	$H_{1} = H_{0} \left( {\frac{{1 - {\text{exp}}\left( { - b_{1} T_{1} } \right)}}{{1 - {\text{exp}}\left( { - b_{1} T_{0} } \right)}}} \right)^{{\left( {b_{2} + \frac{{b_{3} }}{{X_{0} }}} \right)}}$^50^
M4	$H = \frac{{T^{2} }}{{a_{1} + a_{2} T + a_{3} T^{2} }}$	$a_{2} = X$	$X_{0} = \frac{{T_{0} }}{{H_{0} }} - \frac{{b_{1} }}{{T_{0} }} - b_{2} T_{0}$	$H_{1} = \frac{{T_{1}^{2} }}{{b_{1} + X_{0} T_{1} + b_{2} T_{1}^{2} }}$^51^
M5	$H = a_{1} exp\left( { - a_{2} T^{{ - a_{3} }} } \right)$	$a_{2} = X$	$X_{0} = - {\text{ln}}\left( {\frac{{Y_{0} }}{{a_{1} }}} \right)T_{0}^{{a_{3} }}$	$H_{1} = b_{1} \left( {\frac{{H_{0} }}{{b_{1} }}} \right)^{{\left( {\frac{{T_{1} }}{{T_{0} }}} \right)b_{2} }}$^52^

Where* H* is the tree height*, S* site index, *X* and *X*_*0*_ theoretical variable representing site conditions, *T* tree age*, H*_*1*_ is the measured height at age *T*_*1*_, *H*_0_ is a site parameter denoting stand height at age *T*_0_, and *a*_*1*_, *a*_*2*_, *a*_*3*_, *a*_*4*_, *a*_*5*_, *b*_*1*_, *b*_*2*_, and *b*_*3*_ are model parameters.

Local (site-specific) parameters of individual growth series and global parameters of SI models (M1-M5) were simultaneously fitted using the dummy variable approach (DVA), also known as the varying parameter method^53^. In the DVA, global and site-specific parameters are simultaneously estimated, resulting in the same parameter estimates regardless of the selected base age; therefore, it is a base-age invariant method and produces unbiased estimates for base-age invariant equation^54^. The DVA uses a starting value of height as the site-specific parameter for each growth series, assuming all particular growth series measurements have the same starting value. The dynamic model takes the following form Eq. (4).

4 $$H = f\left[ {\sum\limits_{i = 1}^{n} {\alpha_{SIi} d_{i} ;\,b;\,T_{j} ,T_{0} } } \right] + \varepsilon_{ij}$$

where *H* is tree height, *α*_*SIi*_ is the site-specific parameter for tree *i*, whose starting value for fitting is the observed *SI* value at the specified base age, *d*_*i*_ is a dummy variable (being 1 for tree i or 0 for other trees), *b* is a vector for global parameters, *T*_*j*_ is any age, *T*_*0*_ is an initial condition, and *ε*_*ij*_ is the error term. As a result of parameter estimation, an expression containing simulated variables and a site-specific parameter allows the calculation of a unique site parameter for each tree. Due to the longitudinal nature of growth series data, subsequent height observations from the same growth trajectory are significantly correlated, and the assumption of independent errors may be violated. Therefore, the error structured modelling approach with a linear k-order autoregressive error was used in parameter estimation (Eq. 5).

5 $$\varepsilon_{ij} = \varphi_{1} \varepsilon_{ij - 1} r_{1} + \cdots + \varphi_{k} \varepsilon_{ij - k} r_{k} + u_{ij}$$

where *ε*_*ij*_ is the error observed at the *j*th measurement on *i*th tree, *φ*_*k*_—are the *k* parameters to be estimated for the autoregressive process of order *k*, *r*_*k*_ is a dummy variable equal to 1 when *j* > *k* and equal to zero, when *j* < *k*, and *u*_*ij*_ is random noise.

The quality of the fit was evaluated using MAE, ${\text{R}}_{{{\text{adj}}}}^{2}$, and AIC. The criteria mentioned above, and the evaluation of the residuals homoscedasticity, which was carried out based on the distribution of residual values against the predicted values, were used to select the function best fitted to empirical data.

The potential improvement of height growth prediction was analysed by expanding the height growth model parameters as a function of regions. For the calculation of the full model, taking into account the specific effect of region, the vector of global *i*th model parameters (*b*_*i*_) was expanded by the expansion term (*δ*_*iRj*_), which is specific for the given *j*th region (*Rj*) and ith global parameter (*i*). Six dummy variables (*Rj*) representing regions were used to simultaneous fitting the expansion terms specific for each *i*th parameter (*b*_*i*_) and *j*th region.

The significance of the expansion terms was tested using t-statistics and was assumed as a statistical test for the difference between a given global parameter *b*_*i*_ and the regional parameter values. Regional parameter values are the sum of the global parameters *b*_*i*_ and expansion term (*δ*_*iRj*_). Moreover, to compare the fits of the full (regional) and reduced (global) model, we used the anova() function with the regression objects as two separate arguments^41^. The anova() function takes model objects as arguments and returns an ANOVA to check that a more complex model is much better at capturing data than a simpler model.

The full model trajectories were directly compared by plotting the differences between observed and model trajectories. Parameter estimation was carried out in R using the procedures and suitably defined model form^45^.

## Results

### Nonlinear mixed-effects models

The range (0.359–0.710, Table 3) of the mean absolute error indicates that the application for the mixed-effects modelling approach when elaborating the height growth models allows obtaining high accuracy. The obtained results indicate that a better fit characterises the functions with defined two random parameters than one random parameter. The Chapman–Richards function with parameters *a* and *b*, which refer respectively to the asymptote and the growth rate, defined as random effects, was characterised by the best fit (Table 3). Note the shape of the function is expressed as a parameter *c* is estimated as a fixed-effect.

**Table 3:** Adjusted coefficient of determination (${\text{R}}_{{{\text{adj}}}}^{2}$), the Akaike information criterion (AIC) and mean absolute error (MAE) for Chapman-Richards function with different random parameters on both: trees and natural forest regions as grouping levels (random effects are expressed with index i).

Model name	Model type	${\text{R}}_{{{\text{adj}}}}^{2}$	AIC	MAE
Chapman-Richards	$H = a_{i} \left( {1 - e^{bA} } \right)^{c}$	0.985	43,037	0.710
	$H = a\left( {1 - e^{{b_{i} A}} } \right)^{c}$	0.988	40,323	0.643
	$H = a_{i} \left( {1 - e^{bA} } \right)^{{c_{i} }}$	0.995	32,205	0.419
	$H = a_{i} \left( {1 - e^{{b_{i} A}} } \right)^{c}$	0.996	28,711	0.359
	$H = a\left( {1 - e^{{b_{i} A}} } \right)^{{c_{i} }}$	0.995	31,589	0.406

Moreover, analysis of the residuals for the best fitted Chapman-Richards model (estimated parameters shown in Table 4) indicated heteroscedasticity (Fig. 4). The assumed variance function did not model heteroscedasticity well. Likewise, the likelihood test indicated that the variance function has a nonsignificant influence on model fit. Alike autoregressive correlation structure of order 1—AR(1) autoregressive–moving average models for the within-group errors ARMA(10,0) has significant influence on model fit (*p* < 0.0001 for likelihood test). Whereas ARMA(10,0) solution allows to reach the best goodness-of-fit measures (Table 4).

**Table 4:** The parameter estimates and standard errors (in brackets) of the best fitted mixed-effects Chapman–Richards model with (Chapman-Richards AR(1), AR(10) and without (Chapman-Richards)) autoregressive correlation structure.

	Parameter	Chapman–Richards	Chapman–Richards AR(1)	Chapman–Richards ARMA(10,0)
	a	33.838 (1.051)	33.786 (1.025)	33.905 (1.114)
	b	− 0.023 (0.001)	− 0.023 (0.001)	− 0.022 (0.001)
	c	1.432 (0.004)	1.414 (0.007)	1.408 (0.008)
Natural region level	sd(β_1_)	2.444	2.403	2.621
	sd(β_2_)	0.003	0.003	0.003
	corr(β_1_, β_2_)	0.432	0.511	0.510
Tree level	sd(β_1_)	7.095	5.601	5.344
	sd(β_2_)	0.007	0.006	0.006
	corr(β_1_, β_2_)	0.422	0.342	0.254
Residual	–	0.516	0.705	0.617

### Nonlinear fixed-effects models

All preselected functions showed a good fit to the data and explained (in most cases) close to 99% (adjusted R^2^) of the height growth variation (Table 5). The error structured modelling with the 10-order autoregressive error was used in parameter estimation. All developed models prediction errors were relatively low, with root mean absolute error (MAE) varying from 0.59 to 0.69 m.

**Table 5:** Parameter estimates and goodness-of-fit statistics for the individual functions.

Function	Parameter	Estimate	${\text{R}}_{{{\text{adj}}}}^{2}$	AIC	MAE
M1	b1	1.363	0.990	34,961	0.59
	b2	5920.904			
	b3	30.443			
M2	b2	0.017	0.986	38,784	0.69
	b3	1.236			
M3	b1	0.019	0.988	36,536	0.63
	b2	0.864			
	b3	8.293			
M4	b1	10.854	0.989	35,275	0.60
	b2	0.018			
M5	b0	102.076	0.989	36,155	0.63
	b2	0.434			

${\text{R}}_{{{\text{adj}}}}^{2}$ adjusted coefficient of determination, *AIC* akaike information criterion, *MAE* mean absolute error.

Considering the largest part of the explained variance, the smallest average absolute error and the AIC value, the function M1 was selected as best fitted to the data. Next function M1 was used to develop the model taking into account the regional variability. For fitting of the full model, taking into account the specific effect of region, the parameters of the model M1 were expanded as a function of regions (Eq. 6).

6 $$H_{1} = H_{0} \frac{{T_{1}^{{\left( {b_{1} + \delta_{{1R_{j} }} \times Rj} \right)}} \left( {T_{0}^{{\left( {b_{1} + \delta_{{1R_{j} }} \times Rj} \right)}} R + \left( {b_{2} + \delta_{{2R_{j} }} \times R_{j} } \right)} \right)}}{{T_{0}^{{\left( {b_{1} + \delta_{{1R_{j} }} \times Rj} \right)}} \left( {T_{1}^{{\left( {b_{1} + \delta_{{1R_{j} }} \times Rj} \right)}} R + \left( {b_{2} + \delta_{{2R_{j} }} \times R_{j} } \right)} \right)}}$$

where $$R = Z_{0} + \left( {Z_{0}^{2} + \frac{{2\left( {b_{2} + \delta_{{2R_{j} }} \times R_{j} } \right)H_{0} }}{{T_{0}^{{\left( {b_{1} + \delta_{{1R_{j} }} \times Rj} \right)}} }}} \right)^{0.5}$$; $$Z_{0} = H_{0} + \left( {b_{3} + \delta_{{3R_{j} }} \times R_{j} } \right)$$.

The extended full (regional) model explains 0.47% more residual variance, while all expansion terms are statistically significant (t < 0.001, Table 6). Besides, the ANOVA result indicates that more complex, the regional model is significantly better (*p* < 0.01) than the reduced (global) model and thus favour the regional model.

**Table 6:** Parameters of the regional model developed using a nonlinear fixed-effects modelling approach. Expansion terms (δ_iRj_) expressing the difference in the values of ith parameters (b_i_, i = 1, 2, 3) of the Eq. (5) in jth regions (Rj, j = 1, 2, … 6).

Global parameter	Regional expansion term	Estimate	Standard error (m)	t-statistic	p value
1.363	δ_1R1_	− 0.13666	0.004	− 33.2668	< 0.001
	δ_1R2_	0.06231	0.003	21.1719	< 0.001
	δ_1R3_	− 0.09902	0.004	− 24.0611	< 0.001
	δ_1R4_	0.02643	0.006	4.143	< 0.001
	δ_1R5_	0.02700	0.008	3.332	< 0.001
	δ_1R6_	0.08085	0.003	28.843	< 0.001
5920.904	δ_2R1_	25,305.5	1747.008	14.4851	< 0.001
	δ_2R2_	7828.3	441.195	17.7434	< 0.001
	δ_2R3_	31,820.2	1648.675	19.3005	< 0.001
	δ_2R4_	21,248.2	2898.633	7.3304	< 0.001
	δ_2R5_	− 2595.9	339.176	− 7.6536	< 0.001
	δ_2R6_	15,746.3	617.851	25.4855	< 0.001
30.443	δ_3R1_	− 29.0509	2.711	− 10.7145	< 0.001
	δ_3R2_	− 2.8214	0.56	− 5.0352	< 0.001
	δ_3R3_	− 45.0508	2.555	− 17.6339	< 0.001
	δ_3R4_	− 50.8217	6.588	− 7.7144	< 0.001
	δ_3R5_	6.2408	0.766	8.1472	< 0.001
	δ_3R6_	− 39.6201	1.263	− 31.3782	< 0.001

**Figure 2:**
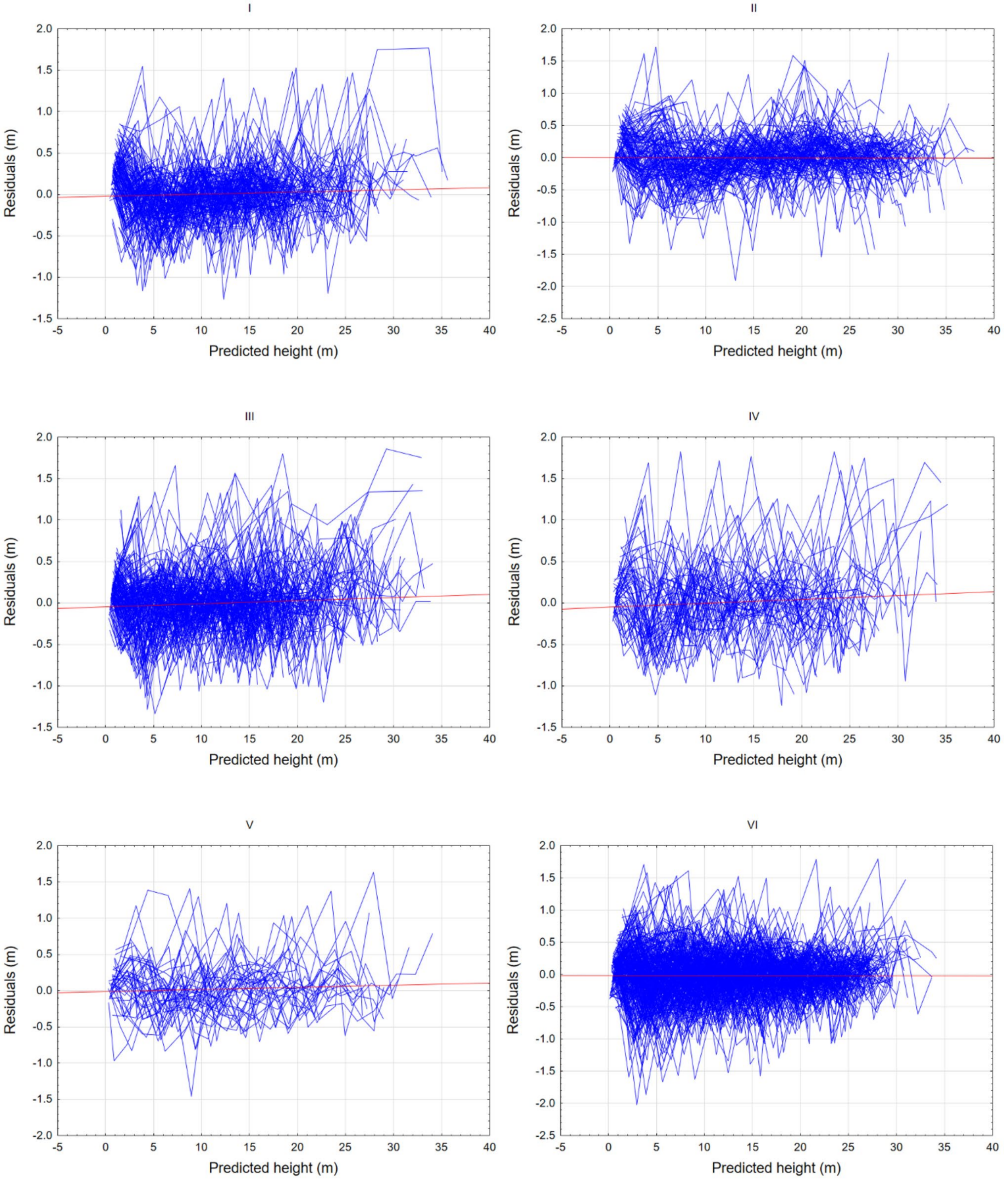
Relationship between residual and predicted height using the site index model for Scots pine in Poland developed using nonlinear fixed-effect modelling approach with the parameters expanded as a function of regions (I–VI).

The residuals of the developed regional growth model are homoscedastic in the entire range of values predicted for individual trees and in individual regions (Fig. 2).

**Figure 3:**
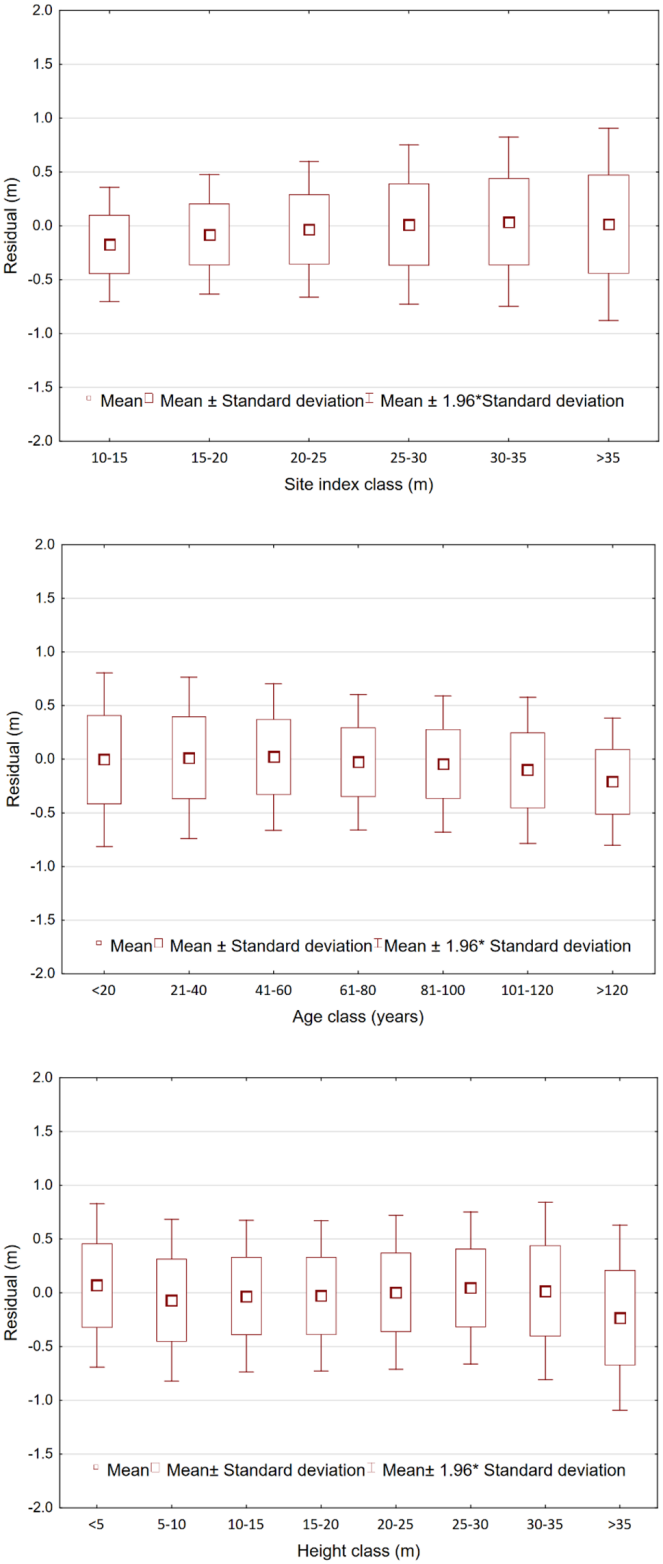
Mean values of the residuals of the regional nonlinear fixed-effect model in site index, age and height classes.

The developed regional model does not cause systematic errors in SI classes, age classes and tree height classes (Fig. 3). The mean residuals slightly deviate from 0 only in the least common SI below 15 m, and the equally rare age class above 120 years and the height class above 35 m (Fig. 3).

### Comparison of regional models developed with the use of nonlinear mixed-effects and nonlinear fixed-effects modelling approach

**Figure 4:**
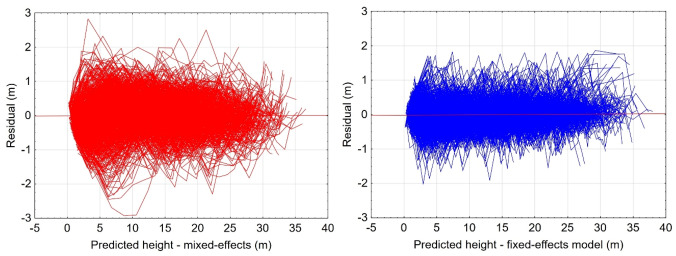
Plot of residuals vs predicted value of height for regional models developed using mixed-effects (left), and fixed-effects (right) modelling approaches.

Based on the model fit statistics and graphical error analysis, it can be concluded that the regional model developed using the fixed-effects approach is characterised by better fit statistics (Table 7) and the distribution of errors (Fig. 4).

**Table 7:** Characterisation of the fit statistics of the regional models developed using mixed-effects and fixed-effects modelling approaches.

Modelling approach	RMSE [m]	MAE	${\text{R}}_{{{\text{adj}}}}^{2}$	AIC
Mixed-effects	0.5596	0.4186	0.9954	15,765
Fixed-effects	0.3698	0.2690	0.9987	12,721

### Analysis of regional variability of height growth patterns based on a model developed using the fixed-effects modelling approach

**Figure 5:**
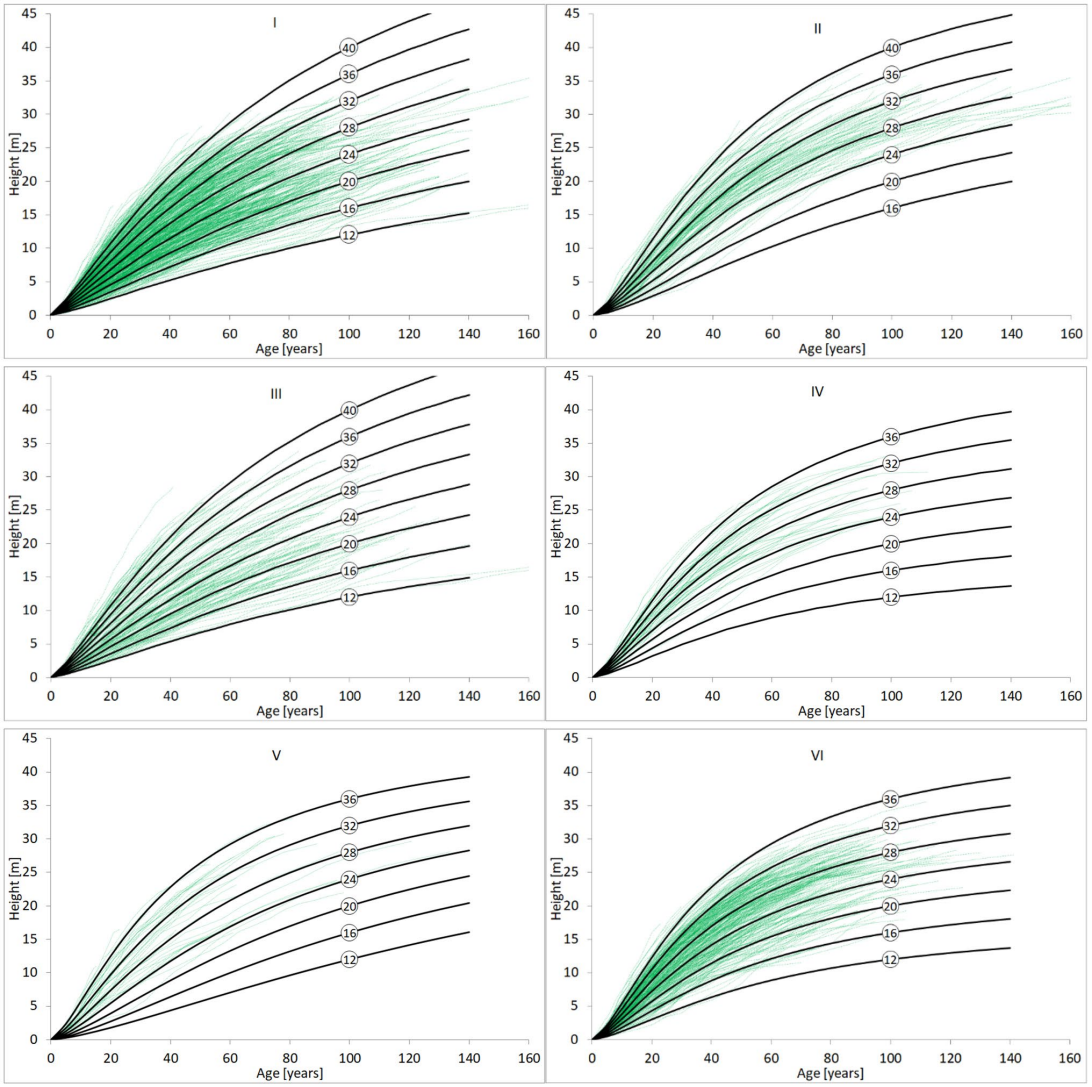
Regional nonlinear fixed-effects model height trajectories against the observed growth series from stem analysis collected in natural forest regions I-VI.

The height growth patterns in individual regions show that it changes directionally from the north and northwest to south and southeast Poland (Fig. 5). In the NFR located in the northwestern (I, III) and northern (II) lowlands of Poland, the height increment rate is also maintained in older stands. In the case of NFR located in the highlands of southern and southeastern Poland, the height increment in old age is inhibited, and therefore the growth curves are more asymptotic (Fig. 5).

**Figure 6:**
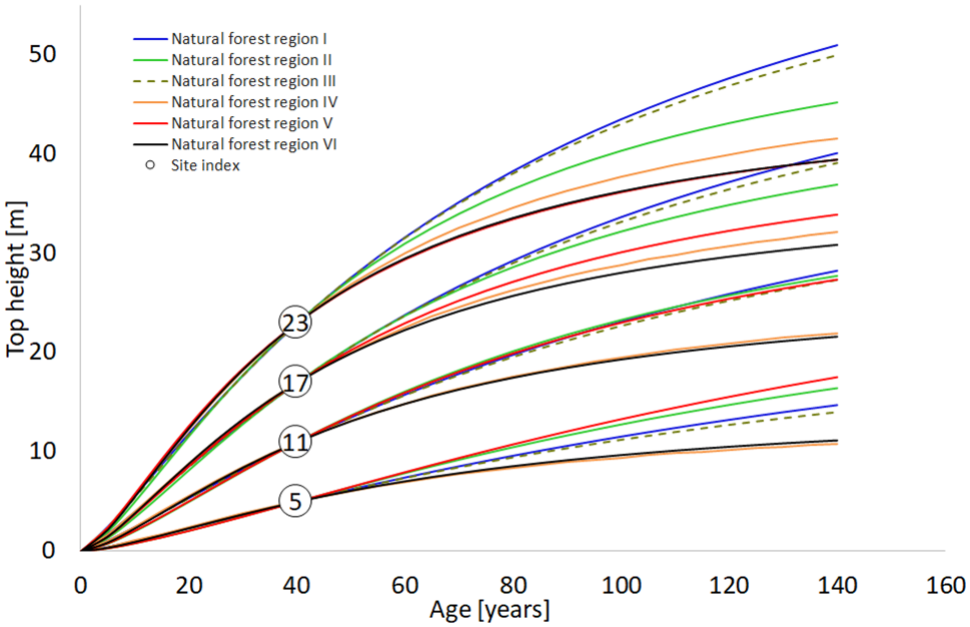
Top height trajectories estimated from the regional fixed-effects model for Scots pines in six natural forest regions (base age 40 years).

Growth trajectories of models developed for individual regions were directly compared using the height at base age 40 as the reference level (Fig. 6). A direct comparison of the height growth trajectories for different SI values determined for the base age of 40 years shows a significant diversification of the height growth patterns between regions, especially in more fertile sites. Up to the base age of 40, the height growth in individual regions is very similar. However, above the base age, a considerable diversification of the growth pattern is observed, especially for most fertile sites with SI equal to 23 or 17 m. Intensive height increment persists for the longest in older stands in the lowlands of northwest and northern Poland, especially in NFR I and III, which are under the influence of the Atlantic climate. Scots pine NFR II located in northeastern Poland grows only slightly more slowly. In the stands growing in regions VI and IV located in the highlands of southeastern Poland, where the climate is continental, the growth is slowed down in older stands. Scots pines from the NRF V located in southern Poland lowlands show weaker growth in old age in the most fertile sites, but in the poorer, their growth dynamics persist until old age (Fig. 6).

## Discussion

We compared the development of regional height growth models using nonlinear fixed-effects and nonlinear mixed-effects modelling approaches. Our results indicate a better fit to the data of the model developed using the nonlinear fixed-effects modelling approach. Comparison of fixed-effects and mixed-effects models was conducted by Nigh^40^ who also found a better fit of models built with GADA. Similar results were also obtained by Cieszewski^55^, who demonstrated that self-referencing GADA models based on the nonlinear fixed-effects approach possess all the desirable properties associated with logical behaviour of the model and estimation statistics, while the nonlinear mixed-effects models lack the well-behaved model properties. The reason for the better fit of the models may be that the nonlinear fixed-effect models ar by definition more flexible than the nonlinear mixed-effect models^55^. Moreover, models can significantly overestimate variances based on untenable assumptions and have more restrictions imposed regarding the site effects' distributional properties. Cieszewski^55^ points out that this is because the NME model fitting process imposes an additional constraint in that the random effect must conform to the arbitrary assumption of randomness, meaning that it must always have a greater or equal sum of square errors when the same models are fit into the same dataset as site models. Our results also confirm well-behaved properties of the nonlinear fixed-effects self-referencing models and their better suitability for modelling TH growth and SI compared to mixed-effects models.

The developed models allowed us to compare the differences in Scots pine height growth dynamics between natural forest regions in Poland. We found that height growth is regionally differentiated. The most relevant differences in growth patterns are observed from the northwest to the southeast gradient. The shape of the growth trajectory for stands located in natural forest regions from the north and northwestern Poland (I–III) differs for younger and older trees. In these natural forest regions, the stands also reach higher absolute heights than those of southern regions. Our results show that the use of the regional model Poland reduces errors in the height growth prediction.

Regional variation in growth patterns was previously reported; these variations have usually been interpreted as the result of variability in climate, geology, soil type, geographical location, and genotype^13–15^.

We found significant differences between the height growth patterns from NRF located in lowlands of northern- northwestern and northeastern Poland and highlands of southern- southeastern Poland. This may be because natural forest regions in northern Poland are under the influence of Atlantic climate and have deep podsolic, brown podsolic, and brown glacial soils, whereas natural forest regions VI and IV are under the influence of continental climate and grow on upland soils with less depth. The depth and physical properties of soil and the sand and clay contents lead to varying soil fertility and productivity^56,57^. Additionally, the availability of water is strongly correlated with the growth of stands and impacts the productivity of sites^58^. A linear increase in net primary production with increased water availability was observed at dry sites^59^. The amount of annual precipitation in Poland changes from south to north and, with soil fertility and depth, can explain the difference in height growth variability between the northern and southern regions.

We found that growth trajectories for individual regions vary with age. The growth of stands in southern and southeastern Poland is slowing rapidly, whereas northern and northeastern stands have more continuous rapid height growth. The difference in growth pattern in regions IV, V, and VI observed in older age can be explained as the impact of soil depth. When soil depth is not a limiting factor in the youngest age classes, the impacts of climate and other factors are more important for growth dynamics, but with the transition to older age classes and increased interspecific competition, soil depth likely begins to limit the growth dynamic. An evident slowdown of growth dynamics in oldest age classes in natural forest regions IV and VI may be the result of a combination of shallower and less fertile podsolic and brown podsolic soils and low annual rainfall. In regions, I, II, and III, geological and climatic conditions are more favourable for Scots pines and likely result in a larger height increment in the older stands.

The significant height growth differences in different regions indicate that the use of one generalised height growth model for the entire country may result in the inappropriate estimation of site productivity and have direct forest management consequences. The importance of regional models may increase because the projections of climate change in Poland predict accentuated differences in site conditions between the northwest and southeast regions^60^. The obtained results concerning the spatial variability of Scots pines' height growth dynamics should be considered when making forest management decisions concerning the rotation age or intensity of silvicultural treatments.

The amount of carbon stored in forests is more significant than in any other terrestrial ecosystem^61^, and tree growth data play a significant role in carbon sequestration and biomass production calculations^62^. Several conversion methods have been developed for different types of forest to obtain reliable estimates^63–65^. However, our results show that not considering specific regional growth conditions and the resulting differentiated growth patterns by using only global models for these calculations may result in an over- or under-estimation of regional height growth, biomass production, and carbon sequestration. Many studies on the estimation of biomass production and carbon sequestration emphasise that the regional approach is promising^66,67^; therefore, the use of models developed on a regional scale shows particular importance for effective forest management.

With the development of geographical information systems and technologies, tools such as the mapping SI are increasingly common^68^. Many problems regarding the stability of forest stands and disturbance of forests in Europe concern relatively small areas. Numerous studies found that SI mapping and analyses, (particularly on a small scale) provide information for a more dynamic forest growth assessment and should be an essential component of regional planning and management^69,70^. Therefore the use of advanced forestry solutions focused on smaller areas, regional growth models, and the analysis of local trends have great potential for the fast detection of problems and the precise formulation of answers to stand instability and forest disturbances.

We used the height growth trajectories of dominant Scots pines collected using the unified methodology to develop a regional model; however, collection methods and data processing in different regions should be carefully executed. Differences in methodology or data sources can lead to significant inconsistencies between growth models that are not based on ecological or biological causes^18^. A given species growth patterns in different ecological or geographical conditions are significant and should be determined before developing local models for small areas. The differences in local model predictions may not be large enough in small geographical regions to justify the development costs^18^. In this situation, smaller regions with low climatic and geomorphological variability should be grouped to develop a single model for larger areas.

## Conclusions

We compared the development of regional height growth models using nonlinear-fixed-effects and nonlinear-mixed-effects modelling approaches. Our results indicate a slightly better fit to the data of the model using the nonlinear fixed-effects approach. The developed models show differences in height growth patterns of Scots pines in Poland and revealed that acknowledgement of region as the independent variable could improve the growth prediction and quality of the SI estimation. The presented study showed regional differences in height growth patterns of Scots pines in Poland. Differences in climate and soil conditions that distinguish natural forest regions affect Scots pine height growth patterns. Therefore, extending this research to models that directly describe height growth interactions with site variables, such as climate, soil properties, and topography, can provide valuable forest management information.

## Data Availability

The datasets used and/or analysed during the current study are available from the corresponding author on reasonable request.

## References

[1] Bontemps JD, Bouriaud O (2014). Predictive approaches to forest site productivity: Recent trends, challenges and future perspectives. Forestry.

[2] Skovsgaard JP, Vanclay JK (2008). Forest site productivity: a review of the evolution of dendrometric concepts for even-aged stands. Forestry.

[3] Albert M, Schmidt M (2010). Climate-sensitive modelling of site-productivity relationships for Norway spruce (*Picea abies* (L.) Karst) and common beech (*Fagus sylvatica* L.). For. Ecol. Manag..

[4] Véga C, St-Onge B (2009). Mapping site index and age by linking a time series of canopy height models with growth curves. For. Ecol. Manag..

[5] Hägglund B, Lundmark JE (1977). Site index estimation by means of site properties of Scots pine and Norway spruce in Sweden. Stud. For. Suec..

[6] Johansson T (1999). Site index curves for common alder and grey alder growing on different types of forest soil in Sweden. Scand. J. For. Res..

[7] Raulier F, Lambert M-C, Pothier D, Ung C-H (2003). Impact of dominant tree dynamics on site index curves. For. Ecol. Manag..

[8] Corral Rivas JJ, Álvarez González JG, Ruíz González AD, von Gadow K (2004). Compatible height and site index models for five pine species in El Salto, Durango (Mexico). For. Ecol. Manag..

[9] Tewari VP, Rivas JJC, VilČko F, Von Gadow K (2007). Height-Growth and site index equations for social forestry plantations of acacia nilotica and eucalyptus hybrid in gujarat state of India. For. Trees Livelihoods.

[10] Monserud RA, Rehfeldt GE (1990). Genetic and environmental components of variation of site index in inland douglas-fir. For. Sci..

[11] Alvarez-González JG, Ruiz-González AD, Rodríguez-Soalleiro R, Barrio-Anta M (2005). Ecorregional site index models for *Pinus pinaster* in Galicia (northwestern Spain). Ann. For. Sci..

[12] Bravo-Oviedo A, Tomé M, Bravo F, Montero G, del Río M (2008). Dominant height growth equations including site attributes in the generalised algebraic difference approach. Can. J. For. Res..

[13] Johansson T (1995). Site index curves for Norway spruce plantations on farmland with different soil types. Stud. For. Suec..

[14] Adams JP, Matney TG, Land SB, Belli KL, Duzan HW (2006). Incorporating genetic parameters into a loblolly pine growth-and-yield model. Can. J. For. Res..

[15] Buford MA, Burkhart HE (1987). Genetic improvement effects on growth and yield of loblolly pine plantations. For. Sci..

[16] Monserud RA (1984). Height growth and site index curves for inland Douglas-fir based on stem analysis data and forest habitat type. For. Sci..

[17] García O (2010). Dynamical implications of the variability representation in site-index modelling. Eur. J. For. Res..

[18] Calama R, Cañadas N, Montero G (2003). Inter-regional variability in site index models for even-aged stands of stone pine (*Pinus pinea* L.) in Spain. Ann. For. Sci..

[19] Adame P, Hynynen J, Cañellas I, del Río M (2008). Individual-tree diameter growth model for rebollo oak (*Quercus pyrenaica* Willd.) coppices. For. Ecol. Manag..

[20] Bravo-Oviedo A, del Río M, Montero G (2007). Geographic variation and parameter assessment in generalised algebraic difference site index modelling. For. Ecol. Manag..

[21] Bontemps J, Bouriaud O (2013). Predictive approaches to forest site productivity: recent trends, challenges and future perspectives. Forestry.

[22] Claessens H, Pauwels D, Thibaut A, Rondeux J (1999). Site index curves and autecology of ash, sycamore and cherry in Wallonia (Southern Belgium). Forestry.

[23] Karlsson K (2000). Height growth patterns of Scots pine and Norway spruce in the coastal areas of western Finland. For. Ecol. Manag..

[24] Martín-Benito D, Gea-Izquierdo G, del Río M, Cañellas I (2008). Long-term trends in dominant-height growth of black pine using dynamic models. For. Ecol. Manag..

[25] Dyrekcja Generalna Lasów Państwowych, *Raport o stanie lasów w Polsce* (2018).

[26] Socha J (2012). Long-term effect of wetland drainage on the productivity of Scots pine stands in Poland. For. Ecol. Manag..

[27] Bravo F, Montero G (2001). Site index estimation in Scots pine (*Pinus sylvestris* L.) stands in the High Ebro Basin (northern Spain) using soil attributes. Forestry.

[28] Gadow, K.; Hui, G. *Modelling Forest Development*, Vol. 57, Forestry Sciences (Springer Netherlands, Dordrecht, 1999). ISBN 978–1–4020–0276–2.

[29] Schwappach A (1943). Ertragstafeln der Wichtigeren Holzarten.

[30] Szymkiewicz, B. Niektóre zagadnienia dotyczące tablic zasobności drzewostanów sosnowych. *Pr. IBL**Seria A* 67 (1948)

[31] Szymkiewicz, B. *Tablice Zasobności i Przyrostu Drzewostanów* (Państwowe Wydawnictwo Rolnicze i Leśne, Warszawa, 2001). ISBN 83–09–01745–6.

[32] Pretzsch H, Biber P, Schütze G, Uhl E, Rötzer T (2014). Forest stand growth dynamics in Central Europe have accelerated since 1870. Nat. Commun..

[33] Socha J, Orzeł S (2013). Dynamic site index curves for Scots pine (*Pinus sylvestris* L.) in southern Poland. Sylwan.

[34] Socha J, Tymińska-Czabańska L, Grabska E, Orzeł S (2020). Site index models for main forest-forming tree species in Poland. Forests.

[35] Esri Inc. ArcGIS Pro (Version 2.2.0). Esri Inc. https://www.esri.com/en-us/arcgis/products/arcgis-pro/overview (2020).

[36] Caudullo G, Welk E, San-Miguel-Ayanz J (2017). Chorological maps for the main European woody species. Data Br..

[37] Zielony, R., Kliczkowska, A. *Regionalizacja Przyrodniczo-Leśna Polski 2010* (2012). ISBN 9788361633624.

[38] Carmean WH (1972). Site index curves for upland oaks in the central states. For. Sci..

[39] Mehtätalo L, Lappi J (2020). Biometry for Forestry and Environmental Data: with Examples in R.

[40] Nigh G (2015). Engelmann spruce site index models: a comparison of model functions and parameterisations. PLoS ONE.

[41] Pinheiro J., Bates D., & DebRoy S. S. D. *nlme: Linear and Nonlinear Mixed Effects Models* (2020).

[42] Wang M, Borders BE, Zhao D (2008). An empirical comparison of two subject-specific approaches to dominant heights modeling: the dummy variable method and the mixed model method. For. Ecol. Manag..

[43] Bronisz K, Mehtätalo L (2020). Mixed-effects generalised height–diameter model for young silver birch stands on post-agricultural lands. For. Ecol. Manage..

[44] Wang M, Bhatti J, Wang Y, Varem-Sanders T (2011). Examining the gain in model prediction accuracy using serial autocorrelation for dominant height prediction. For. Sci..

[45] R Core Team. *R: A Language and Environment for Statistical Computing*. R Foundation for Statistical Computing, Vienna, Austria. URL http://www.R-project.org/ (2020).

[46] Bailey RL, Clutter JL (1974). Base-age invariant polymorphic site curves. For. Sci..

[47] Cieszewski J, Bailey L (2000). Generalized algebraic difference approach: theory based derivation of dynamic site equations with polymorphism and variable asymptotes. For. Sci..

[48] Cieszewski CJ (2001). Three methods of deriving advanced dynamic site equations demonstrated on inland Douglas-fir site curves. Can. J. For. Res. For..

[49] Krumland B, Eng H (2005). Site index systems for major young-growth forest and Woodland species in North California. Calif. For..

[50] Cieszewski CJ (2003). Developing a well-behaved dynamic site equation using a modified hossfeld IV function Y 3 = (axm)/(c + x m–1), a simplified mixed-model and scant subalpine fir data. For. Sci..

[51] Sharma RP, Brunner A, Eid T, Øyen B-H (2011). Modelling dominant height growth from national forest inventory individual tree data with short time series and large age errors. For. Ecol. Manag..

[52] Anta MB, Dorado FC, Diéguez-Aranda U, Álvarez González JG, Parresol BR, Soalleiro RR (2006). Development of a basal area growth system for maritime pine in northwestern Spain using the generalised algebraic difference approach. Can. J. For. Res..

[53] Cieszewski, C. J., Harrison, M. & Martin, S. W. Examples of practical methods for unbiased parameter estimation in self-referencing functions. In *Proceedings of the First International Conference on Measurements and Quantitative Methods and Management and The 1999 Southern Mensur* (ed. Cieszewski, C. J.) (2000).

[54] Nunes L, Patrício M, Tomé J, Tomé M (2011). Modeling dominant height growth of maritime pine in Portugal using GADA methodology with parameters depending on soil and climate variables. Ann. For. Sci..

[55] Cieszewski CJ (2018). Comparing properties of self-referencing models based on nonlinear-fixed-effects versus nonlinear-mixed-effects modeling approaches. Math. Comput. For. Nat. Sci..

[56] Seynave I, Gégout J-C, Hervé J-C, Dhôte J-F, Drapier J, Bruno É, Dumé G (2005). Picea abies site index prediction by environmental factors and understorey vegetation: a two-scale approach based on survey databases. Can. J. For. Res..

[57] Brandl S, Mette T, Falk W, Vallet P, Rötzer T, Pretzsch H (2018). Static site indices from different national forest inventories: harmonisation and prediction from site conditions. Ann. For. Sci..

[58] Breda N, Huc R, André Granier ED (2006). Temperate forest trees and stands under severe drought: a review of ecophysiological responses, adaptation processes and long-term consequences. Ann. For. Sci..

[59] Loik ME, Breshears DD, Lauenroth WK, Belnap J (2004). A multi-scale perspective of water pulses in dryland ecosystems: climatology and ecohydrology of the western USA. Oecologia.

[60] Kundzewicz, Z. W., Hov, Ø., Okruszko, T. Zmiany klimatu i ich wpływ na wybrane sektory w Polsce. 257 (2017).

[61] Barrio Anta M, Dieguez-Aranda U (2005). Site quality of pedunculate oak (*Quercus robur* L.) stands in Galicia (northwest Spain). Eur. J. For. Res..

[62] Bowman DMJS, Brienen RJW, Gloor E, Phillips OL, Prior LD (2013). Detecting trends in tree growth: not so simple. Trends Plant Sci..

[63] Isaev A, Korovin G, Zamolodkchikov D, Utkin A, Pryaznikov A (1995). Carbon stock and depostion in phytomass of the Russian forests.

[64] Fang JY, Wang ZM (2001). Forest biomass estimation at regional and global levels, with special reference to China’s forest biomass. Ecol. Res..

[65] Schroeder P, Brown S, Mo J, Birdsey R, Cieszewski C (1997). Biomass estimation for temperate broadleaf forests of the United States using inventory data. For. Sci..

[66] Bravo-Oviedo A, Gallardo-Andrés C, del Río M, Montero G (2010). Regional changes of *Pinus pinaster* site index in Spain using a climate-based dominant height model. Can. J. For. Res..

[67] Woodbury PB, Smith JE, Weinstein D, Laurence J (1998). Assessing potential climate change effects on loblolly pine growth: a probabilistic regional modeling approach. For. Ecol. Manag..

[68] Latta, G., Temesgen, H. & Barrett, T. M. B. M. Mapping and imputing potential productivity of Pacific Northwest forests using climate variables. Can. J. For. Res. **39**, 1197–1207 (2009).

[69] Coops NC, Hember R, Waring RH (2010). Assessing the impact of current and projected climates on Douglas-Fir productivity in British Columbia, Canada, using a process-based model (3-PG). Can. J. For. Res..

[70] Coops NC, Coggins SB, Kurz W (2007). a Mapping the environmental limitations to growth of coastal Douglas-fir stands on Vancouver Island, British Columbia. Tree Physiol..

